# Regulation of Long Non-Coding RNAs by Statins in Atherosclerosis

**DOI:** 10.3390/biom11050623

**Published:** 2021-04-22

**Authors:** Diamantis I. Tsilimigras, Sofia-Iris Bibli, Gerasimos Siasos, Evangelos Oikonomou, Despina N. Perrea, Konstantinos Filis, Dimitrios Tousoulis, Fragiska Sigala

**Affiliations:** 1 First Propaedeutic Department of Surgery, Division of Vascular Surgery, Hippokration Hospital, National and Kapodistrian University of Athens, 11527 Athens, Greece; kfilis@hotmail.com (K.F.); drfsigala@yahoo.gr (F.S.); 2 Centre for Molecular Medicine, Institute for Vascular Signalling, Goethe University, 60323 Frankfurt am Main, Germany; Bibli@vrc.uni-frankfurt.de; 3 First Department of Cardiology, Hippokration Hospital, National and Kapodistrian University of Athens Medical School, 11527 Athens, Greece; gsiasos@med.uoa.gr (G.S.); boikono@gmail.com (E.O.); drtousoulis@hotmail.com (D.T.); 4 Laboratory for Experimental Surgery and Surgical Research “N.S. Christeas”, National and Kapodistrian University of Athens, 15772 Athens, Greece; dperrea@med.uoa.gr

**Keywords:** statin, RNA, epigenetics, vascular biology

## Abstract

Despite increased public health awareness, atherosclerosis remains a leading cause of mortality worldwide. Significant variations in response to statin treatment have been noted among different populations suggesting that the efficacy of statins may be altered by both genetic and environmental factors. The existing literature suggests that certain long noncoding RNAs (lncRNAs) might be up- or downregulated among patients with atherosclerosis. LncRNA may act on multiple levels (cholesterol homeostasis, vascular inflammation, and plaque destabilization) and exert atheroprotective or atherogenic effects. To date, only a few studies have investigated the interplay between statins and lncRNAs known to be implicated in atherosclerosis. The current review characterizes the role of lncRNAs in atherosclerosis and summarizes the available evidence related to the effect of statins in regulating lncRNAs.

## 1. Introduction

Atherosclerosis is a chronic, progressive disease characterized by the hardening and thickening of the arterial wall and the formation of plaques that consist of immune and mesenchymal cells, lipids, and extracellular matrix [1]. Despite increased public health awareness, atherosclerosis remains a leading cause of mortality worldwide, with an increasing projected prevalence over the next decade [1,2]. High cholesterol levels and particularly low-density lipoprotein cholesterol (LDL-C) increase the risk of atherosclerosis and chronic heart disease [3]. Early treatment of hypercholesterolemia and appropriate control of cholesterol levels represent an important strategy to reduce mortality and morbidity rates associated with cardiovascular events [3]. The use of statins, inhibitors of HMG-CoA reductase, is nowadays the main therapeutic strategy to decrease LDL-C levels [3,4]. Nevertheless, not all patients will achieve physiological cholesterol levels after high-intensity statin treatment [5]. In fact, previous studies have demonstrated significant variations in treatment response among different populations, suggesting that the efficacy of statins may be altered by both genetic and environmental factors [5,6,7].

Long noncoding RNAs (lncRNAs) are transcripts of long RNAs of more than 200 nucleotides that are not translated into proteins [8]. Accumulating evidence shows that lncRNAs essentially contribute to the regulation of gene expression and play an important role in vascular biology, endothelial cell (EC) function, and the development of vascular disease [9,10,11]. The current literature suggests that lncRNAs might be up- or downregulated among patients with atherosclerosis, including coronary artery disease (CAD), peripheral artery disease, or carotid disease [9,10,11]. To date, numerous lncRNAs have been implicated in certain atherogenic processes, including endothelial dysfunction, lipid deposition, and inflammation [9,10,11,12]. In addition, lncRNAs have been demonstrated to be expressed in different cell types known to be implicated in the atherogenic process (e.g., ECs, vascular smooth muscle cells [VSMCs], and macrophages) [9,12]. Although significant progress has been made in characterizing lncRNAs that are implicated in atherosclerosis, it is estimated that fewer than 5% have been identified to date. Furthermore, although still not completely understood, there is evidence to suggest that statins may exert their pleiotropic effects by regulating certain lncRNAs. The present review aims to characterize the current knowledge regarding lncRNAs implicated in the process of atherosclerosis as well as summarize the available evidence related to the effect of statins in regulating lncRNAs.

## 2. LncRNAs in Atherosclerosis: Mechanisms of Action

LncRNAs are present in both the nucleus and cytoplasm and are able to repress and activate genes at the transcriptional as well as post-transcriptional level. Depending on their position, lncRNAs might regulate genes in close proximity (cis) or genes located far away in the genome (trans). LncRNA can be categorized as signaling, decoy, guide, and scaffold lncRNAs depending on their function:

- Signaling lncRNAs act as molecular signals and regulate gene expression via interaction with chromatin-modifying complexes, transcriptional regulators, and DNA.
- Decoy lncRNAs function as decoy molecules that bind to transcriptional regulators and inhibit their interaction with target genes.
- Guide lncRNAs enhance downstream effector functions by helping transcriptional regulators to localize to specific regions.
- Scaffold lncRNAs mediate protein–protein interactions, resulting in the organization of nuclear subdomains, acting as enhancers at certain areas of DNA, or repressing gene expression by creating RNA–DNA structures.

**Figure 1:**
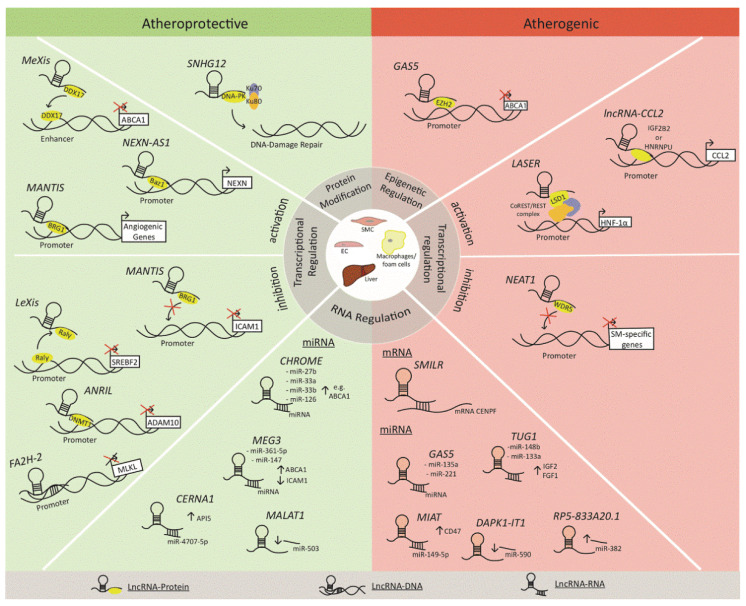
LncRNAs implicated in atherosclerosis (atheroprotective or atherogenic). Reproduced from Josefs et al. *Curr. Atheroscler. Rep.* **2020**, *22*, 55, doi:10.1007/s11883-020-00872-6 [9]; Creative Commons User License. Available online: https://creativecommons.org/licenses/by/4.0 (accessed on 12 April 2021).

Several lncRNAs have been implicated in atherosclerosis (Table 1). Their mechanism of action is somewhat complicated since lncRNA may act on multiple levels and exert atheroprotective or atherogenic effects (Figure 1) [9].

### 2.1. Cholesterol Homeostasis

LncRNAs may regulate cholesterol uptake in liver cells and selectively alter biosynthetic function of lipids in hepatocytes. For example, the lncRNA LASER binds to LSD1 (member of CoREST/REST complex), leading to decreased H3K4me demethylation at the promoter region of the HNF-1α gene, which ultimately increases the expression of PCSK9 in hepatocytes [13]. Given that PCSK9 induces intracellular degradation of low-density lipoprotein receptors (LDLR) [14], the expression of lncRNA LASER is associated with higher circulating cholesterol levels [13]. Another lncRNA implicated in cholesterol metabolism is LeXis, that binds to the ribonucleoprotein Raly, inhibiting its binding to cholesterol biosynthetic gene promoters [15]. In a murine model of familial hypercholesterolemia, gene therapy with adeno-associated virus (AAV8) was utilized to increase LeXis expression, which resulted in reduced lipid accumulation and reduced total cholesterol and triglyceride levels in LeXis-treated mice [16]. In addition, en face lesion analysis revealed decreased atherosclerotic plaques across the aortic root after treatment, suggesting that LeXis might be a potential therapeutic target in atherosclerosis [16].

Another mechanism of cholesterol homeostasis that can be regulated by lncRNAs is the removal of excess cholesterol from circulation and newly formed atherosclerotic plaques via the reverse cholesterol transport pathway. The first step in this process is acquiring cholesterol from peripheral cells via apolipoprotein A1 and HDL through ABCA1 and ABCG1, respectively, followed by delivery to liver and excretion to the bile. The ABC are transmembrane proteins and comprise a superfamily of transporters [17]. The lncRNA MeXis has been shown to amplify liver X receptor (LXR)-dependent transcription of the gene ABCA1 by inducing ABCA1 promoter binding to the transcriptional coactivator DDX17 [18]. In turn, an increased atherosclerotic burden was noted in mice transplanted with MeXis−/− bone marrow as opposed to mice transplanted with wild-type bone marrow after 17 weeks of a Western diet [18]. In contrast to promoting cholesterol efflux, lncRNA GAS5 inhibited ABCA1 expression by binding to its enhancer (EZH2), leading to decreased cholesterol efflux [19]. In turn, GAS5 knockout resulted in decreased progression of atherosclerosis in apoE-deficient mice [19]. Another study demonstrated that the lncRNA CHROME negatively regulated a number of miRNAs (e.g., miRNA miR-27b, miR-33a, miR-33b, and miR-128) in primates [20]. The suppression of these miRNAs led to decreased suppression of the ABCA1 gene, favoring cholesterol efflux and HDL synthesis [20].

### 2.2. Vascular Inflammation

Apart from lipid deposition, inflammation has also been implicated in the initiation and progression of atherosclerosis. One study showed that overexpression of lncRNA NEXN-AS1 suppressed the NF-kB pathway, which led to decreased endothelial cell activation and monocyte recruitment in human vascular endothelial cells (HVEC), thus mitigating atherosclerosis in apoE−/− deficient mice [21]. A subsequent study demonstrated that overexpression of NEXN-AS1 inhibited proinflammatory biomarkers known to drive atherosclerosis (NLRP3, caspase-1, IL-1β, IL-18, etc.) [22]. Another atheroprotective lncRNA is the lncRNA MANTIS, which interacts with the SWI/SNF chromatin remodeling factor BRG1 and inhibits its interaction with the promoter region of monocyte adhesion factor ICAM-1 [23]. MANTIS, in turn, decreased ICAM-1 expression and monocyte adhesion to the activated endothelium suppressing vascular inflammation [23]. Autophagy has also been considered another atheroprotective mechanism [24]. The lncRNA FA2H-2 binds and suppresses the promoter of the MLKL gene, increasing autophagy and preventing macrophages and SMCs from becoming foam cells that favor atherosclerosis [25]. In vitro and in vivo studies demonstrated an anti-inflammatory role of the lncRNA MALAT1 [26,27]. MALAT1 enhanced autophagy in ox-LDL treated HUVEC by sponging miR-216a-5p and upregulating Beclin-1 expression [26]. In addition, lncRNA MALAT1 promoted glucose-induced human endothelial cell pyroptosis [27]. Furthermore, apoE−/− MALAT1−/− mice showed increased plaque formation and infiltration of CD45+ cells compared with apoE−/− MALAT1+/+ mice after consumption of a high-fat diet [28]. Bone marrow cells from apoE−/− MALAT1−/− mice showed increased adhesion to endothelial cells and increased levels of inflammatory mediators [28]. MALAT1 expression was significantly decreased in human plaques compared with normal arteries and was lower in symptomatic versus asymptomatic patients, suggesting a possible atheroprotective role of lncRNA MALAT1 [28].

### 2.3. Plaque Destabilization

Although the majority of atherosclerotic plaques remain clinically silent, chronic inflammation and ongoing monocyte recruitment contribute to plaque growth and, in turn, plaque destabilization, which can lead to life-threatening events such as MI, stroke, embolism. Of note, the lncRNA CCL2 was noted to be increased in unstable symptomatic versus asymptomatic human atherosclerotic plaques [29]. LncRNA CCL2 is a cis-regulatory lncRNA that regulates the expression of CCL2 gene, which encodes monocyte chemoattractant protein 1 that facilitates monocyte recruitment and promotes the progression of vascular inflammation [29]. Another lncRNA shown to promote plaque destabilization is lncRNA NEAT1, which interacts with a chromatin modifier and inhibits expression of smooth muscle cell proteins, facilitating the phenotypic switch of VSMCs from a contractile state to a synthetic state [30]. This switch promotes monocyte recruitment, inflammation, and further plaque destabilization [30]. One example of lncRNA–mRNA interaction is lncRNA SMILR, which regulates mitosis by binding to the mRNA of the mitotic protein CENPF and promotes proliferation of VSMCs [31]. In turn, increased SMILR levels were detected in unstable versus stable human atherosclerotic plaques, suggesting that SMILR might be a potential target limiting vascular remodeling following balloon angioplasty and vessel stenting [31].

## 3. Statins: Mechanism of Action and Rationale for Regulation of LncRNAs

Statins or 3-hydroxy-methylglutaryl coenzyme A (HMG-CoA) reductase inhibitors are powerful tools and widely used drugs in the battle against atherosclerosis [32,33]. As HMG-CoA reductase inhibitors, statins decrease cholesterol biosynthesis and, in turn, decrease serum LDL-C and triglyceride levels [32,33]. Six main statins are currently widely used in clinical practice: pitavastatin, rosuvastatin, atorvastatin, fluvastatin, simvastatin and pravastatin [32]. Apart from LDL-C-dependent functions, statins also demonstrate LDL-C-independent or pleiotropic effects [32]. In fact, statins have been shown to improve endothelial function in a variety of ways, including upregulating eNOS expression through preventing post-translational modification of Rho and activating protein kinase Akt [34,35]. Statins may also regulate vascular oxidative levels and inhibit endothelial inflammatory processes by decreasing leukocyte recruitment [36,37]. Furthermore, previous studies have suggested an attenuation of potent vasoconstrictors typically dysregulated in atherosclerosis, including endothelin-1 and angiotensin-II with statin treatment [38,39].

The chemical properties of statins as inhibitors of HMG-CoA reductase and functional properties as lipid-lowering drugs do not suffice in explaining the majority of their pleiotropic functions. A potential explanation would be the identification of their effects in the transcriptional programs of multiple cell types through the regulation of miRNAs, lncRNAs, or even circular RNAs. Given that increasing evidence suggests the involvement of lncRNAs in atherosclerosis (Table 1, Figure 1), and given that statins exert a number of LDL-C-independent effects, there still remains the question of whether statins exert their pleiotropic functions through regulation of lncRNAs.

## 4. Statins and LncRNAs: Current Evidence

Although treatment with statin represents the main therapeutic strategy against hyperlipidemia and atherosclerosis, there are significant variations in treatment response among different populations [40]. The inter-individual variation in response to statin treatment remains a concern, and the underlying mechanism is not completely understood [40,41]. To date, it is recognized that apart from environmental factors, genetic alterations may also be implicated in the varied efficacy of statins in regulating cholesterol metabolism [40,41,42]. Nevertheless, only a few studies have investigated the interplay between statins and lncRNAs known to be implicated in atherosclerosis (Table 2) [13,22,23,43,44,45,46,47,48].

### 4.1. Cholesterol Homeostasis

Mitchel and colleagues first showed that simvastatin-induced expression of lncRNA RP1-13D10.2 in lymphoblastoid cell lines was higher in high versus low responders [43]. In addition, the study showed that RP1-13D10.2 increased LDLR expression and stimulated LDL uptake in Huh7 and HepG2 cell lines from participants of the Cholesterol and Pharmacogenetics simvastatin clinical trial, suggesting that lncRNAs could potentially contribute to the inter-individual variation in statin response [43]. Another study demonstrated that atorvastatin increased the expression of lncRNA LASER in a dose-dependent manner in HepG2 cells and peripheral blood of patients (patients with no previous statin use that were started on atorvastatin 20 mg/day/5 days) which was accompanied by an increase in PCSK9 both in humans as well as an in vitro model of HepG2 cells [13]. Since PCSK9 has been reported to promote degradation of LDLR [49], this suggested feedback regulation of cholesterol on LASER expression [13]. In contrast to this study, Paez et al. reported that treatment with atorvastatin (20 mg/day/4 weeks) among hypercholesterolemic patients resulted in increased expression of two lncRNAs in peripheral blood samples by RT-qPCR, ARSR and CHROME, but not LASER, among hypercholesterolemic patients, suggesting that statins may differentially regulate the expression of certain lncRNAs [44]. Although a clear explanation for these disparate results does not exist, differences in the duration of treatment (5 days vs. 4 weeks) and the target populations (patients not on statins who started statins for 5 days vs. hypercholesterolemic patients who received statins for 4 weeks) might have accounted for the variations in lncRNA expression with atorvastatin treatment.

### 4.2. Vascular Inflammation

Furthermore, Su et al. reported that treatment with atorvastatin inhibited the expression of lncRNA MEG3 in a hypoxia-induced cardiac progenitor cell (CPC) model [45]. Given that hypoxia inhibits CPC viability and proliferation through modulating MEG3 expression, inhibition of the MEG3/miR22 pathway might be a potential mechanism and target for the development of effective drugs for myocardial repair following myocardial infarction [45]. In another study, atorvastatin was shown to enhance the therapeutic efficacy of mesenchymal stem-cell derived exosomes (MSC^ATV^-Exo) in a rat model of acute myocardial infarction through upregulation of the lncRNA H19 [46]. In fact, silencing lncRNA H19 abolished the cardioprotective effects of exosomes, suggesting that this lncRNA might be, at least in part, responsible for the cardioprotective effect of MSC^ATV^-Exo on infarcted hearts [46]. Wu et al. also demonstrated atorvastatin inhibited pyroptosis via inducing expression of lncRNA NEXN-AS1, suggesting an additional atheroprotective mechanism for statins [22].

More recently, statins have been shown to exert their atheroprotective effects through regulation of the lncRNA MANTIS [11,23]. Indeed, Leisegang et al. showed that certain statins (i.e., cerivastatin, fluvastatin, simvastatin, and atorvastatin) induced the expression of lncRNA MANTIS in both human and cultured endothelial cells. MANTIS had an inhibitory effect on ICAM-1 [11], which is involved in the transendothelial migration of leukocytes to the sites of inflammation [50]. In addition, MANTIS mediated several statin-induced responses, such as regulation of angiogenesis, proliferation, and telomerase activity, to promote endothelial quiescence and vascular protection [11]. Apart from MANTIS, statins also induced lncRNA LISPR1 and SIPR1, resulting in an increased angiogenic capacity required for normal endothelial cell function [47]. Last but not least, a clinical study [51] demonstrated that lncRNA AWPPH—an lncRNA associated with poor prognosis of hepatocellular carcinoma—was highly expressed in patients with coronary artery disease (CAD) when compared with healthy individuals, while it was reduced after treatment with rosuvastatin and atorvastatin, suggesting that AWPPH could be a potential marker to predict outcomes of patients with CAD [48].

Collectively, accumulating evidence has demonstrated that lncRNAs may be implicated in the pleiotropic effects of statins. Despite efforts in identifying the molecular mechanisms of statin treatment, there are only limited data on statins and vascular epigenetics. As such, the present review aimed to summarize the available studies in the field so that investigators can build on this previous work and work towards enhancing our understanding around the mechanisms by which statins exert their pleiotropic effects at the molecular level [52].

## 5. Regulation of LncRNA and Determinants of Statin Efficacy

While the underlying mechanisms of variations in statin response are not completely understood, the association of certain lncRNAs with currently accepted determinants of statin efficacy (i.e., gene polymorphisms, P450 enzyme, efflux and uptake transporters) provides a rationale for further research into how lncRNA regulation might be associated with response to statins. Although only a few studies have directly examined the association of certain statins together with lncRNAs and determinants of statin efficacy to date, the association of the latter two has been more frequently reported.

LncRNAs have emerged as critical players in cellular cholesterol metabolism. Previous studies have reported on the role of lncRNAs LASER, LeXis, MeXis, GAS5, and CHROME in cholesterol metabolism (cholesterol efflux, synthesis etc.) and have been reviewed above [13,15,16,18,19,20]. Cai et al. also reported that overexpression lncRNA ENST00000602558.1 downregulated ABCG1 mRNA and protein expression in VSMCs, leading to decreased ABCG1-mediated cholesterol efflux and increased lipid accumulation [53]. This process might promote VSMC phenotype switching to foam cells, a major mechanism of atherosclerosis [54]. Similarly, Tang et al. also reported that lncRNA ZFAS1 was upregulated in an in vitro model of atherosclerosis (THP-1 macrophage-derived foam cells). Overexpression of ZFAS1 promoted inflammatory responses and decreased cholesterol efflux by upregulating ADAM10/RAB22A expression [55].

In a separate study, Lan et al. identified an lncRNA named lnc-HC that negatively regulated cholesterol metabolism in hepatocytes of an experimental metabolic syndrome rat model [56]. By binding to hnRNPA2B1, the lnc-HC–hnRNPA2B1 complex decreased Cyp7a1 or Abca1 (both on mRNA and protein levels)—both of which are implicated in cellular cholesterol excretion—thus augmenting cholesterol accumulation within hepatocytes [56]. Given that the abovementioned lncRNAs were shown to be important regulators of cholesterol efflux and metabolism, these lncRNAs may represent targets to increase statin efficacy in nonresponders.

In addition, certain lncRNAs have been found to regulate cytochrome P450 [57], and since most statins are metabolized through cytochrome P450, further research is needed to investigate whether certain lncRNAs might be targets for enhancing the response in statin nonresponders.

Genome-wide studies have also demonstrated that combination of certain polymorphisms might be important predictors of statin response [58]. Polymorphisms in lncRNAs have recently been associated with increased risk of cardiovascular events. In particular, Zheng et al. demonstrated that a deletion polymorphism (rs145204276) in the promoter of lncRNA GAS5—implicated in cholesterol efflux and metabolism as noted above [19]—was related to an increased risk of ischemic stroke in humans [59]. Similarly, polymorphisms in lncRNA MEG3 (i.e., rs7158663 and rs4081134) were associated with increased risk of ischemic stroke jointly with polymorphisms in miR-181b rs322931 [60]. In a case–control study, a single nucleotide polymorphism rs4977574 of CDKN2BAS was shown to be a risk factor for coronary heart disease in both females and males under the age of 65 [61]. These results were confirmed in a meta-analysis of 36,452 cases and 39,781 controls which showed that patients with the polymorphism rs4977574 had 27% higher odds of coronary heart disease (OR = 1.27, 95%CI 1.22–1.31) compared with their counterparts [61]. Another case–control study from Pakistan revealed a strong association of polymorphism rs1333049:C > G of lncRNA ANRIL with myocardial infarction [62]. Finally, Li et al. demonstrated that polymorphisms rs9632884 of lncRNA ANRIL and rs3200401 of lncRNA MALAT1 were significantly associated with increased cholesterol and triglyceride levels among both healthy and myocardial infarction patients without necessarily being associated with an increased risk of myocardial infarction [63]. Collectively, given the association of certain lncRNA polymorphisms with a higher frequency of adverse events as well as lipid levels, variations in statin efficacy might be associated with certain lncRNA and other gene polymorphisms.

## 6. Conclusions

LncRNAs can dynamically regulate numerous functions and impact atherosclerotic plaque growth, inflammation, and stability. Accumulating evidence suggests that statins exert their pleiotropic effects, at least in part, by regulating certain lncRNAs. The open question is whether all different classes of statins affect the same pool of regulatory RNA elements (i.e., miRNAs, lncRNAs, or even circular RNAs), resulting in similar transcriptional alterations and enhanced atherosclerosis regulation. This would be unlikely given the heterogenous response of statin receivers. Considering that lncRNAs are emerging as essential mechanisms involved in lipid metabolism and play an important role in controlling transcriptional and post-transcriptional regulatory pathways, lncRNAs represent meaningful candidates to help predict response to statin treatment and explain the variations in treatment response. Although a number of lncRNAs have been implicated in the process of atherosclerosis to date, only a few have been examined in relation to treatment with different classes of statins. Identifying which lncRNA are up- or downregulated with statin treatment is important to formulate targeted therapies for patients with statin-resistant atherosclerosis. Although emerging data have suggested an association between statin treatment and expression of certain lncRNAs, a causal relationship between lncRNA expression and statin efficacy cannot be concluded since findings will need to be confirmed in further animal model studies as well as clinical studies. Integration of currently known determinants of statin efficacy (i.e., gene polymorphisms, P450 enzymes, efflux and uptake transporters etc.) with known and newly detected lncRNAs implicated in atherosclerosis represents a promising field of research. Future studies should map the effects of the statins subclusters in the lncRNA pool to pave the way towards a personalized direction of statin therapy.

## Figures and Tables

**Table 1:** List of lncRNAs implicated in atherosclerosis.

LncRNA	Definition	Humans (Plaques or Serum)	In Vivo (Experimental Models)	In Vitro
LASER	Lipid-associated single nucleotide polymorphism gene region	Positive correlation with Chol levels in PBMC patients	-	Deficiency: ↓ cholesterol in HepG2 cells
LeXis	Liver-expressed LXR-induced sequence	-	Overexpression: C57BL/6 mice, AV LeXis ↓total cholesterol and triglycerides, ↓ aortic root plaques on en face analysis	-
MeXis	Macrophage-expressed LXR-induced sequence	-	Deficiency: Ldlr−/− on WD +MeXis−/− bone marrow↓ Abca1 expression ↑ inflammatory gene expression ↑ lesion size ↑ CD68+ cell	Deficiency: Peritoneal macrophages (MeXis−/− mice fed WD) ↓ ABCA1 ↓ cholesterol efflux ↑ cholesterol accumulation
NEXN-AS1	Nexilin F-actin binding protein antisense RNA 1	↓ atherosclerotic plaques ↓ NEXN in CAD patients (blood)	Deficiency: NEXN± /ApoE−/− on WD ↑ lesion area, macrophage abundance, expression of adhesion molecules ↑ inflammatory cytokines	Overexpression: HUVECs ↓ TLR4/NF-kB pathway ↓ inflammatory gene expression
MANTIS	-	↓ atherosclerotic plaque	Deficiency: Retinal injection of siRNA MANTIS ↑ ICAM-1	Deficiency: HUVECs ↓ angiogenic genes ↑ ICAM-1 ↑ monocyte adhesion ↑ apoptosis ↑ oxidative stress
CCL2	C-C motif chemokine ligand 2	↑ unstable symptomatic atherosclerotic plaque	-	Deficiency: HUVEC (IL-1β) ↓ CCL2
NEAT1	Nuclear paraspeckle assembly transcript 1	-	Deficiency: NEAT1±, carotid artery ligation injury ↓ VSMC proliferation and migration ↓ Neointima formation	Overexpression: ↑ VSMC proliferation and migration Deficiency: ↓ VSMC proliferation and migration
SMILR	Smooth muscle-induced lncRNA enhances replication	↑ unstable atherosclerotic plaque ↑in plasma from patients with high plasma C-reactive protein	-	Deficiency: ↑ Proliferation of arterial and venous SMCs
CHROME	Cholesterol homeostasis regulator of miRNA expression	↑ CAD (plasma), ↑ symptomatic versus asymptomatic atherosclerotic plaques	-	Deficiency: HepG2 cells, primary human hepatocytes, THP-1 ↓ ABCA1 protein expression ↓ cholesterol efflux to exogenous apoA-1
RP5-833A20.1	-	-	Overexpression: ApoE−/− on HFD, LV-induced NFIA OE ↑ cholesterol efflux ↓ lesion size ↓ lipid accumulation	Overexpression: THP-1 (oxLDL) ↓ cholesterol efflux ↑ lipid accumulation ↑ miR-382-5p ↓ NFIA
GAS5	Growth-arrest specific 5	-	Overexpression: ApoE−/− on HFD, LV-induced OE ↓ HDL-C, ↑ LDL-C ↓ reduced cholesterol efflux ↑ lesion size ↑ inflammation	Overexpression: THP-1 (oxLDL) ↓ cholesterol efflux ↑ lipid accumulation ↓ ABCA1 ↑ inflammatory markers ↑ MMP-2, MMP-9 ↑ EZH ↓ miR-135 ↓ miR-221
MALAT1	Metastasis-associated lung adenocarcinoma transcript 1	↓ atherosclerotic plaque, correlates with symptoms of plaque instability	Deficiency: ApoE−/− Malat1−/− bone marrow cells on HFD ↑ adhesion to endothelial cells ↑ proinflammatory mediators ↑ lesion size ↑ miR 503	Deficiency: HUVECs (oxLDL) ↓ autophagy ↑ apoptosis ↑ miR-216a-5p EA.hy926 cells (high glucose) ↓ pryoptosis ↓ NLRP3 ↑ miR-22
MEG3	Maternally expressed 3	-	Overexpression: Ldlr−/− on HFD ↓ CD68+, CD3+, ICAM-1 ↑ collagen content	Overexpression: HMEC-1 ↓ cell viability, migration, tube formation ↑ apoptosis via miR-147 suppression Deficiency: VSMCs ↑ proliferation ↓ apoptosis ↓ ABCA1 via ↓ miR-361-5p suppression
FA2H-2	Fatty acid 2-hydroxylase 2	↓ atherosclerotic plaque	Deficiency: ApoE−/− + LV-si-lncRNA-FA2H-2 on WD ↑ autophagy flux ↑ inflammatory response ↑ increased lesion area	Deficiency: ECs and SMCs (oxLDL) ↑ autophagy flux ↑ increased inflammatory response

Adapted from Josefs et al. *Curr. Atheroscler. Rep.* **2020**, *22*, 55, doi:10.1007/s11883-020-00872-6 [9]; Creative Commons User License. Available online: https://creativecommons.org/licenses/by/4.0 (accessed on 12 April 2021).

**Table 2:** Regulation of lncRNAs by statins.

Author (year)	LncRNA	Definition	Regulation by Statin	Findings	Implications
Mitchel et al. (2016) [43]	RP1-13D10.2	N/A	Simvastatin: Upregulation of RP1-13D10.2 in high responders to statin	-Statin induced expression of RP1-13D10.2 in lymphoblastoid cell lines was higher in the high vs. low responders -RP1-13D10.2 increased LDLR expression and stimulated LDL uptake	RP1-13D10.2 regulates LDLR and may contribute to LDLC response to statin treatment
Li et al. (2019) [13]	LASER	Lipid-associated single nucleotide polymorphism gene region	Atorvastatin: Upregulation of LASER in a dose-dependent manner	-Statin treatment increased LASER expression in HepG2 cells -LASER expression in HepG2 cells was positively correlated with plasma PCSK9 levels in statin-free patients -HNF-1α and PCSK9 were reduced after LASER knockdown in HepG2 cells	Targeting LASER might be an effective approach to enhance the effect of statins
Paez et al. (2020) [44]	ARSR CHROME LASER	ARSR: Activated in renal cell carcinoma (RCC) with sunitinib resistance CHROME: cholesterol homeostasis regulator of miRNA expression LASER: lipid-associated single nucleotide polymorphism gene region	Atorvastatin: Upregulation of ARSR and CHROME	-Statin increased the expression of lncRNAs ARSR and CHROME but not LASER in peripheral blood of hypercholesterolemic patients	Statins differentially regulate the expression of cholesterol-related lncRNAs
Su et al. (2018) [45]	MEG3	Maternally expressed gene 3	Atorvastatin: Downregulation of MEG3	-Atorvastatin protected cardiac progenitor cells (CPCs) from hypoxia-induced injury through inhibiting MEG3 expression -Atorvastatin protected CPCs from hypoxia-induced injury through modulating the MEG3/miR-22/HMGB1 axis.	Molecular mechanism of atorvastatin under hypoxia may provide a target for developing effective drugs for MI patients
Huang et al. (2020) [46]	H19	N/A	Atorvastatin: Upregulation of H19	-MSCATV-Exo resulted in improved recovery in cardiac function and reduced cardiomyocyte apoptosis compared to MSC-Exo. -MSCATV-Exo exhibited a significantly increased level of lncRNA H19 expression. -Silencing lncRNA H19 abolished the cardioprotective effects of MSCATV-Exo in a rat model of acute myocardial infarction	LncRNA H19 might mediate the cardioprotective effects of MSCATV-Exo on acutely infarcted hearts
Wu et al. (2020) [22]	NEXN-AS1	Nexilin F-actin binding protein antisense RNA 1	Atorvastatin: Upregulation of NEXN-AS1 in a dose- and time-dependent manner	-Atorvastatin upregulated lncRNA NEXN-AS1 and NEXN in HUVEC -Atorvastatin inhibited the canonical inflammasome pathway biomarkers of pyroptosis -Inhibition of pyroptosis was diminished by knockdown of lncRNA NEXN-AS1 in HUVEC	Regulation of pyroptosis through lncRNA NEXN might be a potential target against atherosclerosis
Leisegang et al. (2019) [23]	MANTIS	N/A	Cerivastatin, Fluvastatin, simvastatin, atorvastatin: Upregulation of MANTIS	-Statins upregulated lncRNA MANTIS in HUVEC and HAoEC -MANTIS limited the ICAM-1 expression in vivo in mice -expression of MANTIS in human carotid artery endarterectomy material was lower compared with healthy vessels -MANTIS was required to facilitate atorvastatin-induced changes in endothelial gene expression in HUVECs	Strategies to increase lncRNA MANTIS might improve vascular function in nonresponders to statin therapy
Josipovic et al. (2018) [47]	LISPR1	Long intergenic noncoding RNA antisense to S1PR1	Cerivastatin, Fluvastatin, simvastatin, atorvastatin: Upregulation of LISPR1	-LISPR1 was downregulated in EC with vascular pathologies -LISPR1 was induced by statins in HUVECs	LISPR1 might be a potential target for statin-resistant patients
Tang et al. (2020) [48]	AWPPH	LncRNA associated with poor prognosis of hepatocellular carcinoma	Rosuvastatin, atorvastatin: Downregulation of AWPPH	-LncRNA AWPPH was highly expressed in CAD patients -Expression of LncRNA AWPPH was reduced with statin treatment, especially with rosuvastatin in CAD patients	LncRNA AWPPH can be a potential serum marker to predict prognosis of patients with CAD
